# SLAMF7 promotes TCRαβ^+^ double negative T cell antitumor activity through enhancing glutamine metabolism

**DOI:** 10.1186/s13046-025-03570-w

**Published:** 2025-10-30

**Authors:** Nan Xu, Peiyang Fang, Longyang Zhou, Xiaotong Han, Yuan Jiang, Xiyu Wang, Jingjing Zhu, Buer Li, Zihan Zhang, Hua Jin, Xiaonan Du, Guangyong Sun, Dong Zhang

**Affiliations:** 1 Medical Research Center, Beijing Institute of Respiratory Medicine, Beijing Chao-Yang Hospital, Capital Medical University, Beijing, 100020 China; 2 Department of Gastroenterology, Beijing Chao-Yang Hospital, Capital Medical University, Beijing, 100020 China; 3 Liver Research Center, Beijing Friendship Hospital, Capital Medical University, Beijing, 100050 China; 4 General Surgery Department, Beijing Chao-Yang Hospital, Capital Medical University, Beijing, 100020 China; 5 Beijing Laboratory of Oral Health, Capital Medical University School of Basic Medicine, Beijing, 100069 China

**Keywords:** Double negative T cells, SLAMF7, Cancer immunotherapy, ERK, Glutamine metabolism

## Abstract

**Background:**

TCRαβ^+^ double negative T cells (DNT) have recently gained attention for their antitumor activity. Adoptive DNT therapy has emerged as a promising cancer immunotherapy due to its potent cytotoxic function and lack of graft-versus-host-disease. However, the intrinsic mechanisms regulating DNT antitumor functions remain unclear.

**Methods:**

Signaling lymphocytic activation molecule factor 7 (SLAMF7) expression in murine and human DNT were evaluated. The antitumor activities were compared between SLAMF7^+^ and SLAMF7^−^ DNT both *in vivo* and *in vitro*. Further, metabolomics analysis was performed to reveal the underlying mechanism by which SLAMF7 promotes DNT antitumor cytotoxicity.

**Results:**

The expression of SLAMF7 was markedly increased on DNT upon activation. SLAMF7^+^ DNT exhibited superior antitumor capacity both *in vitro* and *in vivo* compared with SLAMF7^−^ DNT. Mechanistically, SLAMF7 enhanced antitumor activity through ligand-independent and ligand-dependent dual manners. Firstly, SLAMF7 could upregulate GPT2/SLC1A5-mediated glutamine metabolism by activating ERK signaling pathway in DNT, thereby supporting mitochondrial fitness, increasing ATP production, enhancing the expression of effector molecules such as granzyme B and perforin, and promoting antitumor activity of DNT against tumor cells independent of homotypic ligand-receptor interactions. Secondly, DNT showed superior antitumor cytotoxicity against SLAMF7-expressing tumor cells because SLAMF7-SLAMF7 interaction between DNT and SLAMF7-expressing tumor cells promoted DNT cell degranulation. Furthermore, SLAMF7 was also highly expressed in human DNT, and its dual antitumor roles in human DNT were also validated.

**Conclusions:**

SLAMF7 is a key regulator of DNT-mediated cytotoxicity and a promising target for improving DNT cell function in cancer therapy.

**Supplementary Information:**

The online version contains supplementary material available at 10.1186/s13046-025-03570-w.

## Significance

SLAMF7 enhances DNT antitumor activity through both ligand-independent and ligand-dependent dual patterns, which can be a potential target for improving cancer immunotherapy.

**Supplementary Information:**

The online version contains supplementary material available at 10.1186/s13046-025-03570-w.

## Introduction

TCRαβ^+^ double negative T cells (DNT) are TCRαβ^+^ lymphocytes that do not express CD4, CD8, or natural killer (NK) cell surface markers. Despite constituting less than 5% of T lymphocytes in peripheral blood and lymphoid tissues, DNT have been reported to play critical roles in antitumor immunity [1, 2]. In both murine and human pancreatic ductal adenocarcinoma, DNT is a prominent population of tumor-infiltrating activated T cells that can slow tumor growth [3]. In murine sarcoma models, innate-like features of DNT are associated with neutrophil-dependent anti-sarcoma immunity [4].

Recently, DNT have garnered increasing interest as a novel adoptive cell therapy (ACT) due to their lack of graft-versus-host-disease (GVHD) and off-the-shelf use [5, 6]. A phase I clinical trial was the first to utilize allogeneic DNT to treat relapsed acute myeloid leukemia post allogeneic hematopoietic stem cell transplantation, showing favorable safety and encouraging antileukemic response [7]. In a preclinical study, adoptive DNT transfer significantly inhibited tumor progression in two transplantable sarcoma models [4]. These growing evidences highlight the therapeutic potential of DNT in cancer, prompting further investigation into their molecular mechanisms.

DNT have been shown to effectively eliminate tumor cells in both hematologic malignancies and solid tumors through multiple pathways, including direct cytotoxicity via IFN-γ, perforin, and granzyme B secretion [4, 8, 9]. Additionally, DNT exert antitumor activity through various ligand-receptor interactions, such as NKG2D/NKG2DL, DNAM-1/DNAM-1L, FasL/Fas, and TRAIL/TRAILR [6, 10]. However, the intrinsic regulatory mechanisms underlying DNT-mediated antitumor responses within the tumor microenvironment remain to be fully elucidated.

Signaling lymphocytic activation molecule factor 7 (SLAMF7), a member of SLAM family receptors, is a homotypic-binding transmembrane receptor (i.e., self-ligand) [11]. It is physiologically expressed on immune cells, including NK cells, activated T cells, B cells, macrophages, dendritic cells (DCs) and plasma cells, and also expressed on the surface of some malignant tumor cells, such as B-cell non-Hodgkin lymphoma (B-NHL) and multiple myeloma (MM) [12–14]. SLAMF7 has been reported to promote the induction and activation of NK cell cytotoxicity [15, 16], and enhance macrophage-mediated phagocytosis of tumor cells [13, 17], recognized as a critical immunoregulatory receptor involved in antitumor immunity [18, 19]. Therefore, it is necessary to explore its functional role in DNT-mediated antitumor responses.

In this study, we demonstrated that SLAMF7 is highly expressed on activated DNT, and not only intrinsically enhances the cytotoxic function of DNT but also engages in homotypic ligand-receptor interactions with SLAMF7-expressing tumor cells, exerting dual effects on DNT antitumor immunity. Mechanistically, SLAMF7 upregulates GPT2/SCL1A5-mediated glutamine metabolism by activating ERK signaling pathway in DNT, thereby promoting cytotoxic molecules expression and antitumor responses.

## Materials and methods

### Cell lines and cell culture

Murine cell lines A20 (B-NHL) and CT26 (colon carcinoma), and human cell lines Raji (B-NHL), Colo205 (colon carcinoma), MHCC97H (hepatocarcinoma) and embryonic kidney HEK 293 T (293T) were obtained from the National Infrastructure of Cell Line Resource (Beijing, China). B-NHL cell lines were cultured in RPMI 1640 (Gibco) containing 10% fetal bovine serum (FBS, Gibco), while colon carcinoma, hepatocarcinoma and 293 T cell lines were maintained in DMEM (Gibco) containing 10% FBS.

### Mice

C57BL/6 (6-8 weeks, male), BALB/c nude (6-8 weeks, male) and BALB/c (6-8 weeks, male) mice were provided by Beijing Vital River Laboratory Animal Technology Co., Ltd, which were bred and maintained under specific pathogen-free conditions. All animal experiments were conducted in accordance with the guidelines of the Institutional Animal Care and Ethics Committee of Beijing Chao-Yang Hospital (20-2042).

### Tumor engraftment

A20 cells in logarithmic growth were resuspended in PBS. For tumor engraftment, 2 × 10^6^ or 1 × 10^6^ A20 cells were injected subcutaneously into the forelimb armpit of BALB/c or BALB/c nude mice, respectively. To assess tumor microenvironment changes, BALB/c mice were euthanized on day 20 post-engraftment, and tumor tissues were collected for analysis. For adoptive transfer models, BALB/c nude mice were randomly divided into three groups on day 13 post-engraftment, 5 × 10^6^ SLAMF7^high^ DNT or SLAMF7^low^ DNT, or PBS (control group) were adoptively transferred to three groups. Meanwhile, recombinant mouse IL-2 (Peprotech, USA) was administered intraperitoneally (10^4^ IU per mouse) every other day from the day of DNT injection until the end of the experiment. Tumor size was measured every 2 days to record the tumor volume and draw the growth curve. Tumor volume (mm^3^) was calculated using the formula: volume = (length × width^2^)/2. At the endpoint, all mice were euthanized for final tumor volume and weight assessment, and tumor tissues were collected for flow cytometric analysis of tumor cells and immune cells.

### Single-cell RNA sequencing analysis

Single-cell RNA sequencing (scRNA-seq) was performed as previously described [20, 21]. Briefly, sorted viable single-cell suspensions were processed using 10× Genomics platform. The sequencing data were subsequently analyzed using Cell Ranger and Seurat R package.

### Expansion of murine DNT *in vitro*

The expansion of DNT was carried out as previously described [20]. In brief, murine natural DNT were sorted from spleen and lymph nodes of mice, and then cocultured with mature DCs and murine recombinant IL-2 (50 ng/mL) for 7 days to obtain activated DNT.

### Expansion of human DNT *in vitro*

Human DNT (hDNT) were isolated from peripheral blood of healthy donors using Double-negative T Cell Isolation Kit (Miltenyi Biotec, Germany), then cultured with 50 ng/ml recombinant human IL-2 (Peprotech) and 2 ug/ml anti-human CD3 antibody for activation and expansion. After 15-20 days, hDNT were collected for experiments. All procedures involving human participants were conducted in accordance with the ethical standards of the Beijing Chao-Yang Hospital Ethical Committee (2024-ke-961).

### SiRNA interference

A20 cells or DNT (1 × 10^6^/well) were respectively seeded in 6- or 12-well plates until they grew to approximately 60% coverage, then transfected with SLAMF7-targeting or control siRNA using siRNA-Mate plus transfection reagent (GenePharma, China) following the manufacturer’s instructions. The targeting sequences are listed in supplementary Table S1. SLAMF7 knockdown efficiency was assessed by quantitative real-time PCR (qRT-PCR).

### Plasmid overexpression

The murine Slamf7 pcDNA3.1-T2A-EGFP and control plasmids, the human wild-type Slamf7, mutant Y284F and Y304F and control plasmids were constructed by YouBio Biological Company (China). CT26 cells were transfected with the plasmids using Lipofectamine 3000 transfection reagent (Invitrogen, USA) following the manufacturer’s protocol. The efficiency of SLAMF7 overexpression was detected through qRT-PCR and western blotting.

### Quantitative real-time PCR (qRT-PCR)

Total RNA was extracted using Trizol reagent (Invitrogen) and reverse-transcribed into complementary DNA (cDNA). qRT-PCR was performed using SYBR Green on a 7500 Fast Real-Time PCR System (Applied Biosystems). β-actin was selected as a control gene for normalization, and relative gene expression was calculated using comparative Ct method. Primer sequences are listed in supplementary Table S1.

### Western blotting

Cells were collected and lysed for protein extracts. Lysates were then subjected to 10% SDS-PAGE and transferred to PVDF membranes. Primary antibodies against p-ERK1/2 (4370; Cell Signaling Technology), ERK1/2 (4695; Cell Signaling Technology), Flag (80801-2-RR, Proteintech) and β-actin (3700; Cell Signaling Technology), and relevant secondary antibodies conjugated to HRP were used.

### Flow cytometric analysis

For *in vivo* experiments in mice, dead cells were excluded using 7-AAD (surface) or Zombie Aqua (intracellular) staining (BioLegend). Lymphocyte populations were identified with anti-mouse CD45 (clone 30-F11), CD3 (17A2), TCRβ (H57-597), CD49b (DX5), NK1.1 (S17016D), CD4 (GK1.5) and CD8a (53 − 6.7) antibodies. Macrophage populations were identified with anti-mouse CD11b (M1/70), F4/80 (BM8), MHC-II (M5/114.15.2), and CD206 (C068C2) antibodies. For both *in vivo* and *in vitro* experiments in mice, DNT phenotypes were assessed using anti-mouse SLAMF7 (4G2), Ki67 (16A8), granzyme A (3G8.5), granzyme B (QA16A02), perforin (S16009A), Annexin V, BCL2 (BCL/10C4), NKG2D (CX5), CD107a (1D4B), CD69 (H1.2F3), GPT2 (16757-1-AP, Proteintech), SLC1A5 (20350-1-AP, Proteintech) and phosphor-ERK1/2 (6B8B69) antibodies. For *in vitro* experiments in human, DNT phenotypes were analyzed with anti-human SLAMF7 (162.1), Ki67 (11F6), granzyme B (QA16A02), perforin (dG9), Annexin V, and CD107a (H4A3) antibodies. The gating strategy is shown in Figure S1A and S1B.

### *In vitro* killing assay

Target cells and DNT were co-incubated for 24 h at appropriate effector-to-target ratios. In parallel, target cells were incubated alone to determine basal lysis. Cells were then stained with appropriate surface antibodies to distinguish target cells from DNT. Annexin V and 7-AAD were stained to detect basal and sample lysis of target cells. The percentage of specific lysis was calculated by the formula: Specific lysis (%) = 100 × (% Annexin V^+^_with DNT_ - % Annexin V^+^_without DNT_)/(100 - % Annexin V^+^_without DNT_).

### Degranulation assay

To assess cell degranulation, DNT were stimulated using antibodies as described previously [15, 16, 22]. In brief, DNT were incubated with anti-SLAMF7 antibody (4G2) for 5 min at 37℃ followed by rabbit anti-rat IgG. Besides, hDNT were incubated with Elotuzumab (HuLuc63) for 24 h at 37℃. Degranulation was then analyzed by quantifying the percentage of DNT expressing CD107a-positive on the cell surface.

### RNA sequencing (RNA-seq) analysis

DNT were sorted into SLAMF7^+^ and SLAMF7^−^ group by flow cytometry, and total RNA was extracted. RNA-seq (Annoroad Gene Technology, China) was performed as we previously reported [23]. In brief, sequencing was performed on Illumina HiSeq2000 platform, and reads were aligned to the mouse genome (Mm9). DESeq2 R package was used to identify differentially expressed genes (DEGs), screened with absolute fold change ≥ 2 and *P*adj value ≤ 0.05. ClusterProfiler R package was used to conduct Gene Ontology (GO) and Kyoto Encyclopedia of Genes and Genomes (KEGG) analysis. Gene set enrichment analysis (GSEA) was performed using the tools provided by OmicStudio (https://www.omicstudio.cn/tool).

### Metabolomics analysis

Targeted metabolomics was performed by Metabo-Profile (Shanghai, China). 5 × 10^6^ cells were rapidly removed into liquid nitrogen for storage. Subsequently, metabolite levels were measured using ultra-performance liquid chromatography coupled to a tandem mass spectrometry (UPLC-MS/MS) system. Raw data were quantitated using MassLynx software (v4.1, Waters Corp) to calculate the concentration of metabolites.

### Glutamine uptake assay

Glutamine uptake assay was measured using Glutamine/Glutamate-Glo™ Assay kit (Promega, USA) according to the manufacturer’s instructions. Cell culture media at the starting time point (T0) and later 24 h (T24) were both collected for measurement of glutamine concentrations. Cellular glutamine uptake levels were calculated by subtracting glutamine concentration at T24 from that at T0.

### Cellular ATP level measurement

Cellular ATP levels were measured using the Enhanced ATP Assay Kit (Beyotime Biotechnology, China) according to the manufacturer’s instructions. Briefly, 1 × 10^5^ cells were transferred to a luminometer plate, lysed with 20 µl buffer, and mixed with 100 µl ATP detection reagent, and the plate was read using BioTek Synergy H1 (Agilent Technologies, USA).

### Gene expression dataset analyses

SLAMF7 expression data used in pan-cancer analysis were obtained from The Cancer Genome Atlas (TCGA) database (https://portal.gdc.cancer.gov). In brief, raw data were download using the TCGAbiolinks (v2.28.4) R packages, and normalized using a log2(TPM + 1) transformation. Data were plotted using the ggplot2 R package (v3.4.4).

These pan-cancer types consisted of bladder urothelial carcinoma (BLCA), breast invasive carcinoma (BRCA), cervical squamous cell carcinoma and endocervical adenocarcinoma (CESC), cholangiocarcinoma (CHOL), colon adenocarcinoma (COAD), esophageal carcinoma (ESCA), head and neck squamous cell carcinoma (HNSC), kidney chromophobe (KICH), kidney renal clear cell carcinoma (KIRC), kidney renal papillary cell carcinoma (KIRP), liver hepatocellular carcinoma (LIHC), lung adenocarcinoma (LUAD), lung squamous cell carcinoma (LUSC), pancreatic adenocarcinoma (PAAD), pheochromocytoma and paraganglioma (PCPG), prostate adenocarcinoma (PRAD), rectum adenocarcinoma (READ), stomach adenocarcinoma (STAD), thyroid carcinoma (THCA) and uterine corpus endometrial carcinoma (UCEC).

### Statistical analysis

All data were presented as mean ± standard deviation (SD). The normal distribution of variables was assessed using the Shapiro-Wilk test. For two-group comparisons, t-test was used for normally distributed data and Mann-Whitney test for non-normal data. For comparisons among more than two groups, One-way ANOVA with post hoc test was used for normal variables, and Kruskal-Wallis test was used for non-normal variables. A value of 0.05 was used as the significance level and statistical significance was indicated as *P < 0.05, **P < 0.01, ***P < 0.001, and not significant (ns, *P* > 0.05), respectively. The statistical analyses were conducted using SPSS version 26.0 (SPSS Inc., Chicago, IL) and GraphPad Prism 9 (GraphPad Software Inc., La Jolla, CA).

## Results

### SLAMF7 expression is upregulated on DNT upon activation and correlates with functional molecule expression

**Fig. 1:**
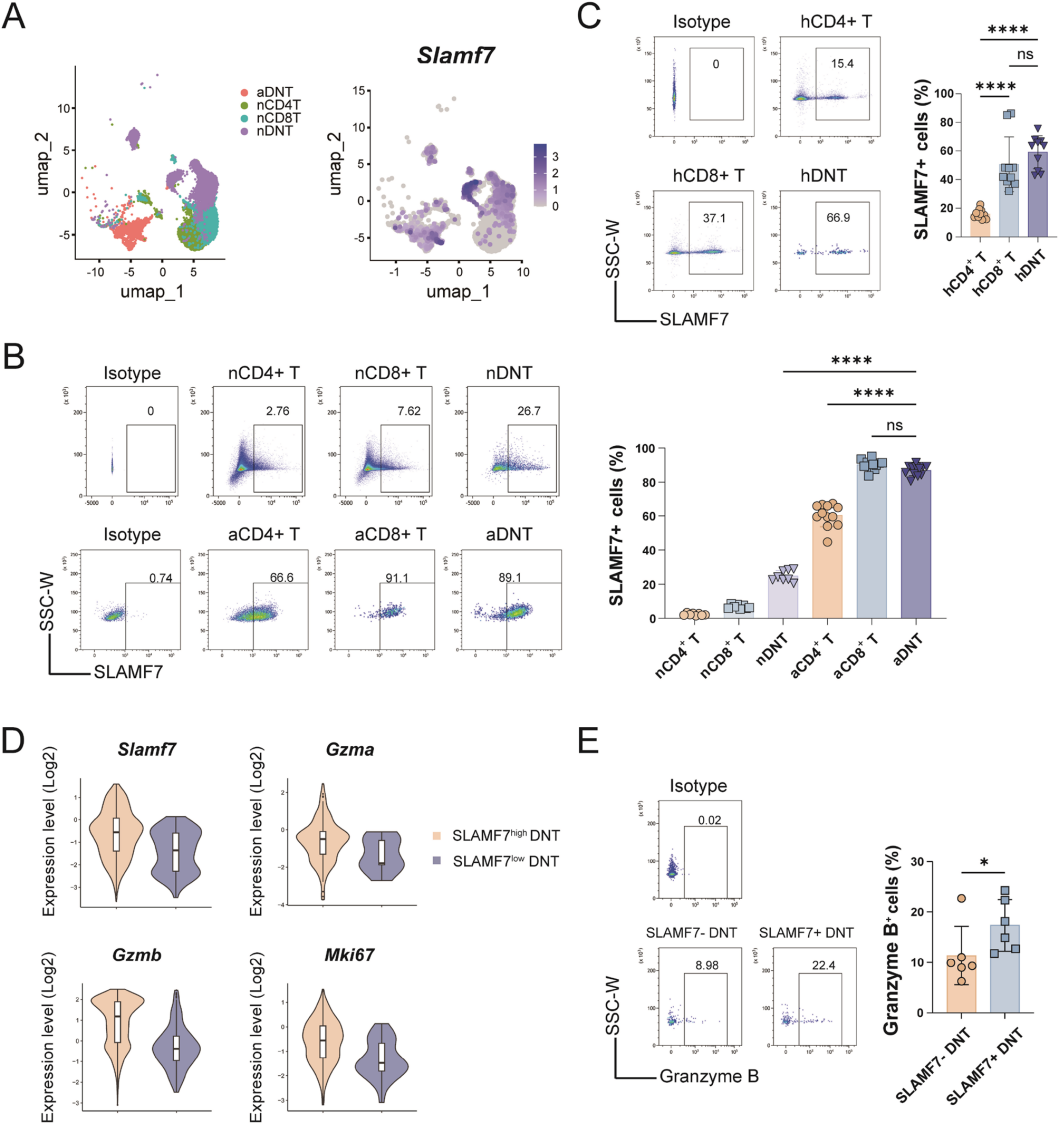
DNT highly expressed SLAMF7, and the expression level is closely related to regulatory function. **A** UMAP analysis of previously published murine scRNA-seq data (GSE129030) showing distribution and *Slamf7* expression in murine DNT and conventional T cell subsets. **B** SLAMF7 expression in murine DNT and conventional T cells tested by flow cytometry. **C** SLAMF7 expression in human PBMC-derived DNT and conventional T cells tested by flow cytometry. **D** The expression levels of *Slamf7*, *Gzma*, *Gzmb* and *Mki67* in SLAMF7^−^ and SLAMF7^+^ DNT subsets from previously published murine scRNA-seq data (GSE129030). **E** Flow cytometric analysis of GZMB expression in SLAMF7^−^ and SLAMF7^+^ tumor-infiltrating DNT from BALB/c mice bearing A20 tumors (*n* = 6)

 By analyzing our previously acquired scRNA-seq data(20 [21]),we found SLAMF7 was predominantly expressed on both naïve DNT (nDNT) and activated DNT (aDNT) rather than naïve CD4^+^ (nCD4^+^) and naïve CD8^+^ (nCD8^+^) T cells (Fig. 1A). Then, we validated these findings by flow cytometry. SLAMF7 was absent on nCD4^+^ T and nCD8^+^ T cells, rarely expressed on naïve DNT. Upon activation, both aDNT and activated CD8^+^ (aCD8^+^) T cells had significantly increased SLAMF7 expression than activated CD4^+^ (aCD4^+^) T cells (Fig. 1B). Expression of SLAMF7 was further determined in T cell subsets isolated from peripheral blood mononuclear cells (PBMC) of healthy controls (Fig. 1C). Similar to the findings in mice, human DNT (hDNT) and human CD8^+^ (hCD8^+^) T cells showed higher levels of SLAMF7 than human CD4^+^ (hCD4^+^) T cells, which suggested that SLAMF7 is an activation-dependent molecule, preferentially expressed in cytotoxic T cell subsets, and conserved across species.

Next, we investigated its cytotoxic-related functions on DNT. Using our previous murine scRNA-seq data [20, 21], we found that SLAMF7^high^ cells exhibited higher levels of *Gzma*, *Gzmb* and *Mki67* than SLAMF7^low^ cells in DNT, indicating that SLAMF7 might serve as a potential intrinsic functional regulator of cytotoxic and proliferative potential in DNT (Fig. 1D). To further explore the relationship between DNT functional molecules and SLAMF7 expression in the tumor microenvironment (TME), we analyzed DNT infiltrating tumors in an implanted A20 tumor model. Notably, SLAMF7^+^ DNT within the TME had a significant higher level of granzyme B than SLAMF7^−^ DNT (Fig. 1E), supporting that SLAMF7 expression is associated with enhanced cytotoxic potential *in vivo*.

### SLAMF7^+^ DNT exhibit a stronger antitumor cytotoxicity *in vivo* and *in vitro*

**Fig. 2:**
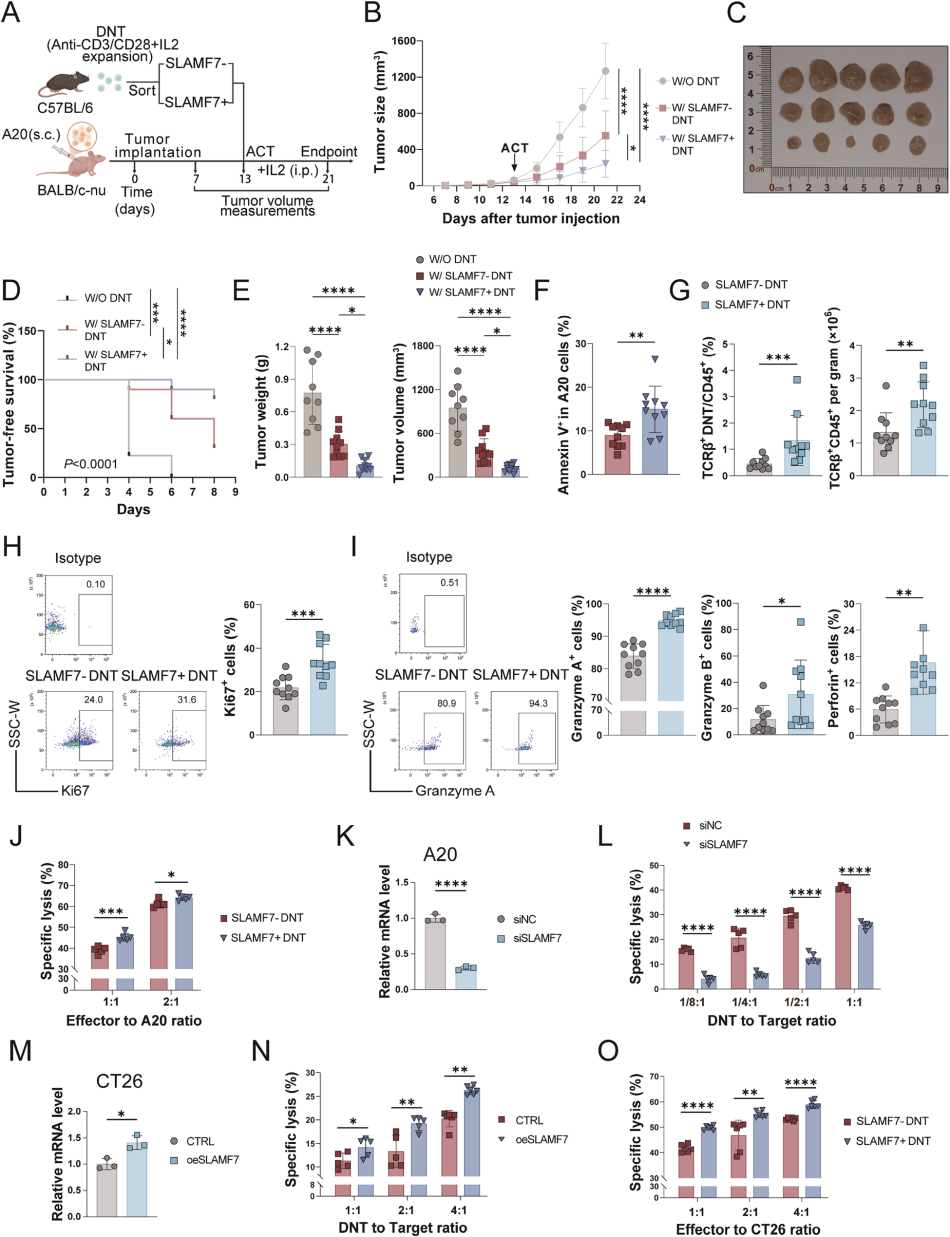
SLAMF7^+^ DNT demonstrate superior antitumor cytotoxicity both *in vivo* and *in vitro*. **A** Schematic of adoptive transfer of SLAMF7⁺ or SLAMF7⁻ DNT into BALB/c nude mice bearing A20 tumors. (PBS control group: *n* = 9; SLAMF7⁻ DNT group: *n* = 10; SLAMF7⁺ DNT group: *n* = 10). **B** Tumor growth curves for tumor-bearing mice treated with PBS, SLAMF7^-^ or SLAMF7^+^ DNT. **C** Representative tumor images. **D** Kaplan-Meier survival analysis showing tumor-free survival (defined as the time from date of treatment to the first appearance of tumor at least 400 mm^3^ in size). **E** Tumor weight and volume measured at endpoint. **F** Apoptotic A20 tumor cells quantified by Annexin V⁺ percentage. **G** Tumor-infiltrating DNT analyzed by flow cytometry (TCRβ⁺CD45⁺); absolute numbers per gram tumor shown. (**H-I**) Flow cytometric analysis of proliferative marker Ki67 **H** and cytotoxic markers GZMA, GZMB and Perforin **I** among transferred DNT in vivo. **J** Specific lysis (calculated as: 100 × (% Annexin V^+^_with DNT_ - % Annexin V^+^_without DNT_)/(100 - % Annexin V^+^_without DNT_)) of A20 cells coincubated with SLAMF7^-^ and SLAMF7^+^ DNT for 24 h in vitro. **K** SLAMF7 knockdown efficiency in A20 cells detected by qRT-PCR. **L** Specific lysis of contorl (siNC) and SLAMF7-knockdown (siSLAMF7) A20 cells coincubated with DNT for 24 h in vitro. **M** SLAMF7 overexpression efficiency in CT26 cells detected by qRT-PCR. **N** Specific lysis of contorl (CTRL) and SLAMF7-overexpressing (oeSLAMF7) CT26 cells coincubated with DNT for 24 h *in vitro*. **O** Specific lysis of CT26 cells coincubated with SLAMF7^-^ and SLAMF7^+^ DNT for 24 h *in vitro.*

To explore whether SLAMF7⁺ DNT possess enhanced antitumor activity, we adoptively transferred either SLAMF7^−^ or SLAMF7^+^ DNT into immunodeficient BALB/c nude mice bearing subcutaneous A20 tumors. After tumor establishment, either SLAMF7^−^ or SLAMF7^+^ DNT from C57BL/6 mice were administered peritumorally after *in vitro* activation (Fig. 2A). Although both SLAMF7^−^ or SLAMF7^+^ DNT administrations markedly suppressed A20 tumor growth, SLAMF7^+^ DNT exhibited more profound tumor growth inhibition (Fig. 2B and C), and Kaplan-Meier analysis revealed that SLAMF7⁺ DNT treatment significantly prolonged tumor-free survival compared to SLAMF7^−^ DNT or PBS treatment (Fig. 2D), also evidenced by final tumor weight and volume (Fig. 2E). Additionally, Flow cytometric analysis revealed that SLAMF7^+^ DNT induced a higher level of tumor cell apoptosis than SLAMF7^−^ DNT (Fig. 2F). Compared to SLAMF7^−^ DNT, adoptively transferred SLAMF7^+^ DNT had an increased tumor-infiltrating level (Fig. 2G), elevated Ki67 expression (Fig. 2H), and significantly elevated production of cytotoxic molecules, such as granzyme A, granzyme B and perforin (Fig. 2I). These findings demonstrated that SLAMF7 enhances DNT-mediated tumor control *in vivo*.

To explore whether SLAMF7⁺ DNT modulate the tumor immune microenvironment, we profiled tumor-infiltrating innate immune populations in BALB/c nude mice. Flow cytometric analysis revealed no significant differences in the frequency or number of intratumoral NK cells across treatment groups (Figure S1C). Similarly, the total macrophage and M1-like macrophage populations remained similar among groups, while the number of M2-like macrophages was significantly reduced in tumors treated with SLAMF7⁺ DNT, suggesting a shift away from an immunosuppressive phenotype (Figure S1D). These findings indicated that SLAMF7⁺ DNT might also modulate the tumor microenvironment by affecting M2-like macrophages.

Previous studies reported that SLAMF7 is thought to engage in homotypic interactions with its own ligand to mediate tumor cell killing [13, 16, 17]. To investigate whether SLAMF7 on DNT contributes to tumor cytotoxicity through this mechanism, we firstly assessed SLAMF7 expression in various tumor cell lines, which showed that A20 cells had the highest SLAMF7 expression, while CT26 cells had no SLAMF7 expression (Figure S2A). When DNT cocultured with A20 cells, SLAMF7^+^ DNT induced significantly higher levels of A20 cell apoptosis than SLAMF7^−^ DNT (Fig. 2J). To further validate the critical role of SLAMF7 ligation in DNT antitumor activity, we knocked down SLAMF7 expression in A20 cells (Fig. 2K), and then co-cultured these cells with DNT. As shown in Fig. 2L, decreased SLAMF7 expression on A20 cells impaired the tumor-killing activity of DNT. Furthermore, we transfected plasmids to SLAMF7 non-expressing CT26 colorectal tumor cells to upregulate their SLAMF7 expression (Fig. 2M). SLAMF7-overexpressing CT26 cells exhibited increased apoptosis than SLAMF7 non-expressing CT26 cells when cocultured with DNT (Fig. 2N). These data suggested that the critical role of SLAMF7 homotypic interactions in DNT-mediated antitumor activity.

Interestingly, when we cocultured DNT with SLAMF7 non-expressing CT26 cells, SLAMF7^+^ DNT also induced significantly a higher level of apoptosis of CT26 cells than SLAMF7^−^ DNT (Fig. 2O), indicating that beyond homotypic ligand-receptor interactions, SLAMF7 might intrinsically enhance DNT antitumor function, thereby exerting a dual role in antitumor immunity.

### SLAMF7 enhances DNT antitumor cytotoxicity in a ligand-dependent manner

**Fig. 3:**
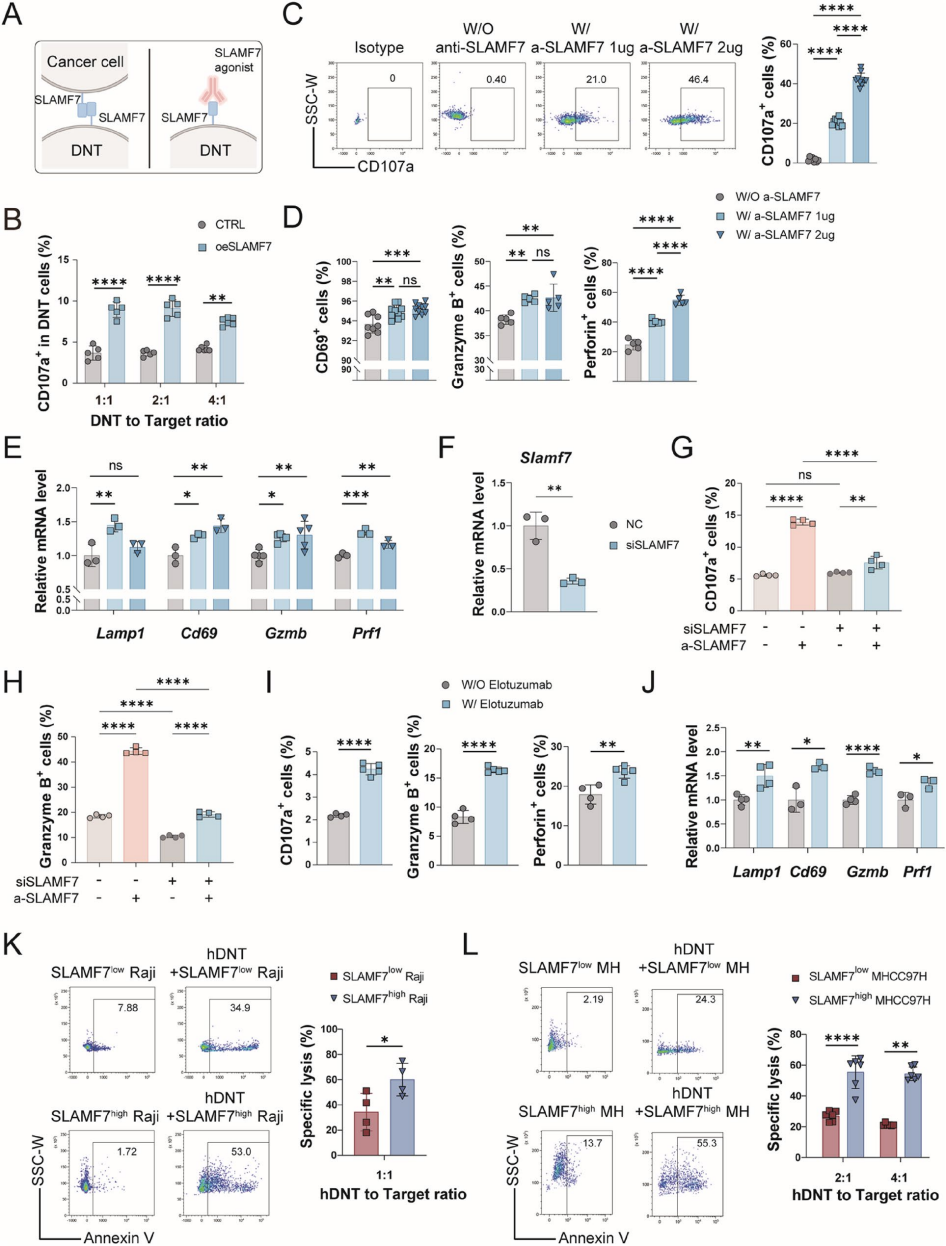
SLAMF7 ligation could enhance antitumor cytotoxicity in DNT through increasing cell degranulation. **A** A schematic of SLAMF7 ligation. **B** CD107a surface expression on DNT after 24 h co-culture with control or SLAMF7-overexpressing CT26 tumor cells. (**C-D**) Flow cytometric analysis of the percentage of DNT expressing CD107a at cell surface **C**, activation marker CD69 and cytotoxic molecules GZMB and Perforin **D** after stimulating or not stimulating with the anti-SLAMF7 and rabbit anti-rat IgG. **E** The mRNA level of *Lamp1*, *Cd69*, *Gzmb* and *Prf1* in DNT stimulated or not stimulated with murine SLAMF7 agonist. **F** SLAMF7 knockdown efficiency in DNT detected by qRT-PCR. (**G-H**) Flow cytometric analysis of CD107a at cell surface **G** and GZMB **H** in control and SLAMF7-knockdown DNT stimulated or not stimulated with murine SLAMF7 agonist. (**I-J**) Expression of CD107a, CD69, GZMB and Perforin in hDNT after stimulating or not stimulating with Elotuzumab tested by flow cytometry **I** or qRT-PCR **J**. (**K-L**) Specific lysis of FACS-sorted SLAMF7^-^ and SLAMF7^+^ Raji cells **K** or MHCC97H cells **L** coincubated with hDNT for 24 h *in vitro*

To better elucidate the role of SLAMF7 in DNT-mediated tumor killing via homotypic ligand-receptor interaction, we performed *in vitro* co-culture experiments and employed an anti-SLAMF7 antibody acting as receptor agonists to mimic ligand engagement (Fig. 3A). Coculture with SLAMF7-overexpressing CT26 tumor cells significantly increased CD107a (Lysosomal-associated membrane protein 1, LAMP1) surface expression on DNT, a maker of degranulation, compared to control CT26 cells, indicating that SLAMF7 ligation could promote DNT cell degranulation (Fig. 3B). Whereas the effect was less pronounced at higher effector-to-target (E: T) ratio (4:1), potentially due to target cell saturation or limited ligand availability. Next, we used anti-SLAMF7 antibody to stimulate DNT. Anti-SLAMF7 antibody stimulation similarly elevated CD107a⁺ DNT frequency (Fig. 3C). Besides, increased expression levels of activated maker CD69 and cytotoxic molecules, granzyme B and perforin were also found in DNT after anti-SLAMF7 antibody stimulation (Fig. 3D). Likewise, the same alterations were observed at mRNA level (Fig. 3E).

To definitively establish that the agonistic anti-SLAMF7 antibody acts through direct receptor engagement, we knocked down SLAMF7 expression in DNT (Fig. 3F) and compared the responses of control and knockdown cells to antibody stimulation. The antibody triggered potent upregulation of CD107a and GZMB in control DNT, but this response was significantly blunted in SLAMF7-knockdown DNT, as assessed by both absolute expression and relative fold change (Fig. 3G and H, S2B). This result confirms that antibody-mediated activation is contingent upon SLAMF7 expression on DNT. Importantly, we also observed that SLAMF7 knockdown intrinsically reduced baseline GZMB levels, uncovering a cell-autonomous, ligand-independent role for SLAMF7 in potentiating the cytotoxic machinery of DNT.

These findings were further validated in human DNT stimulated with or without elotuzumab, a humanized anti-SLAMF7 monoclonal antibody. SLAMF7 engagement with elotuzumab enhanced the expression of CD107a, granzyme B and perforin at both protein and mRNA levels in hDNT (Fig. 3I and J). We also sought to determine if the activation of hDNT by elotuzumab requires Fc-mediated cross-linking. In an Fc-blocking assay, a control human IgG1 exhibited no activating effect. In contrast, elotuzumab robustly induced hDNT degranulation even under Fc receptor blockade (Figure S2C), indicating that SLAMF7 engagement alone is sufficient to enhance hDNT cytotoxicity. These data support a model in which elotuzumab acts as a direct SLAMF7 agonist to promote degranulation. To definitively confirm this agonistic mechanism, future investigations should utilize Fc-inert formats of elotuzumab, such as F(ab’)₂ fragments or Fc-mutant variants. Furthermore, we chose two SLAMF7-expressing tumor cells, a human lymphoma cell line Raji and a human hepatocarcinoma cell line MHCC97H (Figure S2A), sorted on SLAMF7 expression respectively and cocultured with hDNT. As expected, SLAMF7^high^ tumor cells were more effectively killed by hDNT than SLAMF7^low^ tumor cells, either Raji or MHCC97H (Fig. 3K and L). Altogether, these data demonstrated that SLAMF7 ligation could enhance DNT-mediated antitumor cytotoxicity through promoting cell degranulation.

Given the observed SLAMF7-dependent antitumor effects, we next evaluated SLAMF7 expression in paired tumor and adjacent normal tissues based on TCGA’s database to explore the translational potential of SLAMF7⁺ DNT therapy. As shown in Figure S2D, SLAMF7 expression was significantly elevated in tumor tissues from multiple cancers, including head and neck squamous cell carcinoma (HNSC), kidney renal clear cell carcinoma (KIRC), kidney renal papillary cell carcinoma (KIRP), and lung adenocarcinoma (LUAD). The result suggested that SLAMF7⁺ DNT therapy may be more applicable to selected solid tumors.

### SLAMF7 enhances intrinsic cytotoxic function of DNT in a ligand-independent manner

**Fig. 4:**
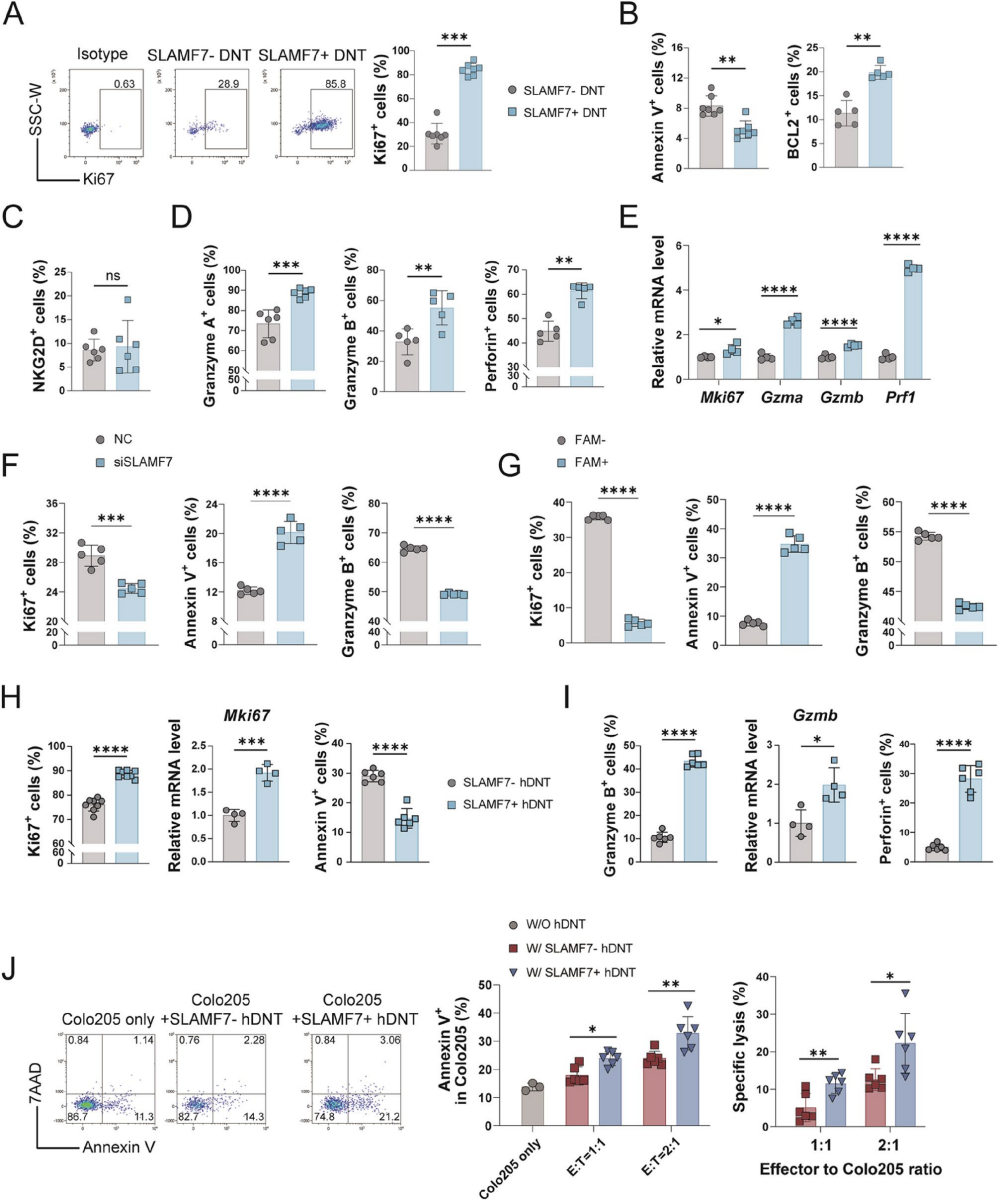
SLAMF7 is essential for DNT intrinsic antitumor function. (**A-D**) Flow cytometric analysis of proliferative marker Ki67 **A**, apoptotic makers **B** and cytotoxic markers NKG2D **C**, GZMA, GZMB and Perforin **D** in SLAMF7^-^ and SLAMF7^+^ DNT in vitro. **E** The mRNA level of *Mki67*, *Gzma*, *Gzmb* and *Prf1* in SLAMF7^-^ and SLAMF7^+^ DNT tested by qRT-PCR. (**F-G**) Flow cytometric analysis of Ki67, Annexin V and GZMB expression in contorl (siNC) and SLAMF7-knockdowned (siSLAMF7) DNT **F**, and in FAM^-^ and FAM^+^ SLAMF7-knockdowned DNT **G**. (**H-I**) The expression level of survival markers **H** and cytotoxic markers **I** in SLAMF7^-^ and SLAMF7^+^ hDNT tested by flow cytometry or qRT-PCR. **J** The percentages of apoptosis and specific lysis of Colo205 tumor cells after 24 h coculture with SLAMF7⁻ or SLAMF7⁺ hDNT *in vitro*

We further examined the role of SLAMF7 as an intrinsic functional regulator of DNT-mediated antitumor responses. SLAMF7⁺ DNT exhibited significantly higher expression of the proliferation marker Ki67 and the anti-apoptotic marker BCL2, accompanied by a reduced apoptosis rate (Fig. 4A and B), confirming that SLAMF7 promotes DNT survival and tumor-infiltrating capacity. In addition, DNT have been reported to effectively kill tumor cells through cytotoxic molecules secretion or NKG2D/NKG2DL pathway [24], we detected whether SLAMF7 is involved in the regulation of these molecules. There was no obvious difference in NKG2D expression between SLAMF7^−^ and SLAMF7^+^ DNT (Fig. 4C), whereas the expression of granzyme A, granzyme B and perforin were significantly higher in SLAMF7^+^ DNT than those in SLAMF7^−^ DNT (Fig. 4D), consistent with the scRNA-seq *and in vivo* results of DNT. Likewise, the mRNA level of *Mki67*, *Gzma*, *Gzmb* and *Prf1* were significantly upregulated in SLAMF7^+^ DNT (Fig. 4E). To ascertain the functional consequences of SLAMF7 loss, we transfected DNT with FAM-labeled siRNA targeting SLAMF7 (Fig. 3F). Bulk analysis of transfected cells revealed that SLAMF7 knockdown reduced Ki67 and granzyme B levels while increasing apoptosis of DNT (Fig. 4F). Crucially, when we compared the FAM⁺ (transfected) and FAM⁻ (non-transfected) subpopulations directly, the FAM⁺ DNT showed significantly greater impairment in proliferation and cytotoxicity, and higher apoptosis (Fig. 4G). This internal control validates that the observed phenotypes are a direct result of SLAMF7 knockdown.

These findings were further validated in human DNT derived from PBMCs. Compared to SLAMF7^−^ hDNT, the mRNA and protein levels of Ki67 were both significantly upregulated and the apoptotic rate was decreased (Fig. 4H), and the expression of cytotoxic molecules mentioned above were also elevated (Fig. 4I) in SLAMF7^+^ hDNT. Furthermore, when cocultured with the SLAMF7⁻ human colorectal cancer cell line Colo205 (Figure S2A), SLAMF7^+^ hDNT induced significantly higher levels of tumor cell apoptosis and more effectively killed tumor cells than SLAMF7^−^ hDNT (Fig. 4J). Taken together, SLAMF7 is essential for DNT cell survival and cytotoxicity, and intrinsic antitumor function in the absence of homotypic ligand engagement.

### SLAMF7 promotes glutamine metabolism in DNT

**Fig. 5:**
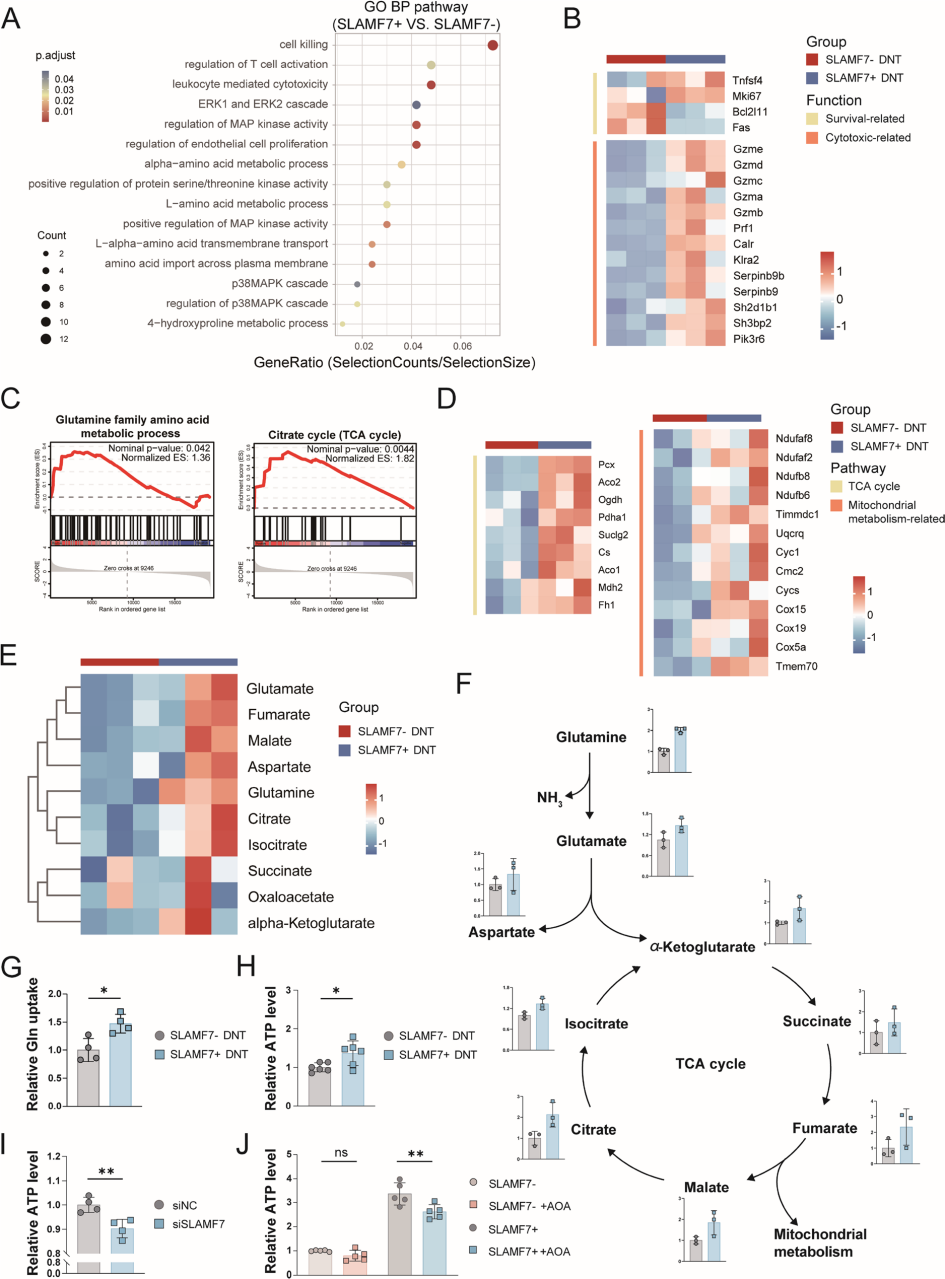
SLAMF7 enhances glutamine metabolism in DNT. (**A-B**) Gene Ontology (GO) enrichment of DEGs **A** and heatmap analysis of survival and cytotoxic-related genes **B** based on RNA-seq data. (**C-D**) GSEA enrichment analysis **C** and heatmap of TCA cycle and mitochondrial metabolism-related genes **D**. (**E-F**) Heatmap showing differentially expressed levels of representative metabolites **E** and schematic of glutamine metabolism **F**. (**G-I**) Effect of SLAMF7 downregulation on glutamine uptake **G** and ATP levels (**H-I**) in DNT. **J** Comparison of ATP levels in SLAMF7^-^ and SLAMF7^+^ DNT after addition of Aminooxyacetic Acid (AOA), an aminotransferase inhibitor

To explore the underlying mechanism by which SLAMF7 intrinsically boosts DNT cytotoxic function in a ligand-independent manner, we performed RNA sequencing (RNA-seq) in DNT with or without SLAMF7 expression. Of the 373 differentially expressed genes (DEGs) identified, 174 were upregulated and 199 were downregulated between SLAMF7^−^ and SLAMF7^+^ DNT (Figure S2E). Biological processes (BP) analysis revealed that DEGs were enriched primarily in T cell activation, cytotoxicity, amino acid metabolic and transmembrane transport process (Fig. 5A). Consistent with the observed *in vivo* and *in vitro* results, the heatmap showed that genes critical for cell survival and cytotoxicity were significantly upregulated in SLAMF7^+^ DNT (Fig. 5B), and the gene set enrichment analysis (GSEA) demonstrated that apoptotic process was significantly downregulated (Figure S2F) but cytolytic granule was significantly upregulated (Figure S2G) in SLAMF7^+^ DNT. Additionally, the related genes were verified by qRT-PCR analysis (Figure S2H).

Importantly, GSEA analysis further identified glutamine family amino acid metabolic process and TCA cycle were significantly upregulated in SLAMF7^+^ DNT (Fig. 5C). Moreover, genes critical for TCA cycle and mitochondrial metabolism were significantly upregulated in SLAMF7^+^ DNT (Fig. 5D), which validated using qRT-PCR (Figure S2I). These findings indicated that SLAMF7 may play a key role in regulating glutamine metabolic process in DNT.

To elaborate the relationship between SLAMF7 expression and glutamine metabolic process, we first performed metabolites profiling in DNT with or without SLAMF7 expression. The heatmap revealed that most metabolites involved in glutamine metabolism and TCA cycle were increased in SLAMF7^+^ DNT (Fig. 5E). Further analysis of the LC-MS data showed that the level of glutamine was elevated, leading to increased intracellular glutamate levels, then glutamate undergoes transamination with oxaloacetate to generate aspartate and α-ketoglutarate, the latter of which enters the TCA cycle, the overall levels of most glutamine-derived amino acids and TCA cycle intermediates were increased in SLAMF7^+^ DNT (Fig. 5F), thereby enhancing mitochondrial metabolism and supporting increased metabolic activity [25]. Furthermore, we observed an increase of glutamine uptake in SLAMF7^+^ DNT (Fig. 5G). Consistent with the elevated level of TCA cycle intermediates, SLAMF7^+^ DNT exhibited a higher level of ATP production than SLAMF7^−^ DNT, confirming that glutamine metabolism was more active in SLAMF7^+^ DNT (Fig. 5H). Similar result was obtained by SLAMF7 knockdown in DNT, with reduced ATP production (Fig. 5I). Inhibition of glutamine metabolism by aminooxyacetic acid (AOA), an aminotransferase inhibitor [26], significantly inhibited ATP production in SLAMF7^+^ DNT but had no significant effect on SLAMF7^−^ DNT (Fig. 5J), these confirmed that SLAMF7 promotes glutamine metabolism in DNT.

### SLC1A5 and GPT2 are targets of glutamine metabolism pathway regulated by SLAMF7 in DNT

**Fig. 6:**
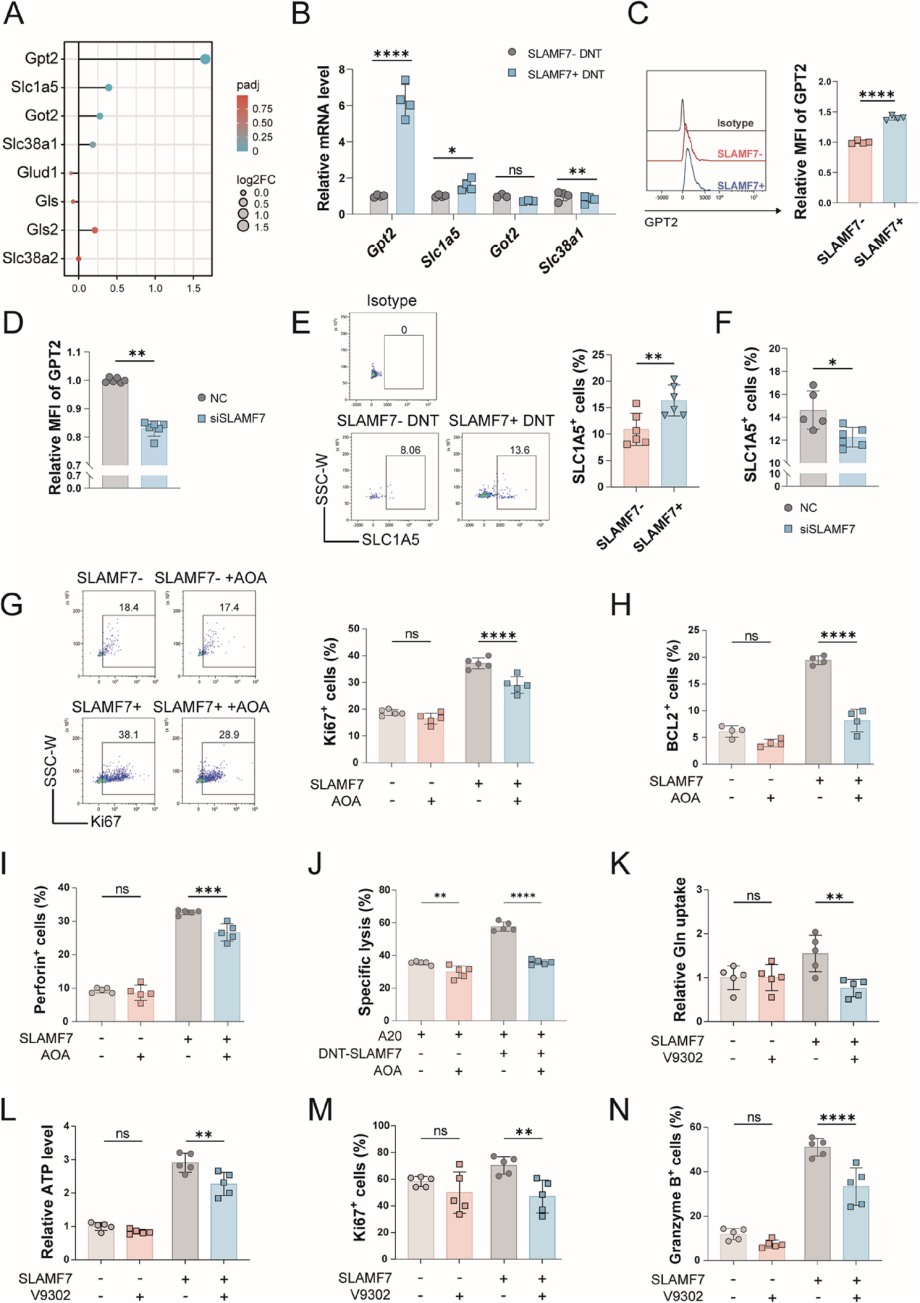
SLC1A5 and GPT2 are targets of SLAMF7-regulated glutamine metabolism in DNT. (**A-B**) Differential expression of glutamine metabolism-related genes in SLAMF7^+^ DNT compared to SLAMF7^-^ DNT based on RNA-seq data **A** and validated by qRT-PCR **B**. (**C-D**) Flow cytometric analysis of GPT2 expression in SLAMF7^-^ and SLAMF7^+^ DNT **C** or in contorl (siNC) and SLAMF7-knockdown (siSLAMF7) DNT **D**. (**E-F**) Flow cytometric analysis of SLC1A5 expression in SLAMF7^-^ and SLAMF7^+^ DNT **E** or in siNC and siSLAMF7 DNT **F**. (**G-I**) Flow cytometric analysis of the expression of Ki67 **G**, BCL2 **H** and Perforin **I** in SLAMF7^-^ and SLAMF7^+^ DNT after addition of AOA. **J** Specific lysis of A20 tumor cells coincubated with SLAMF7⁺ or SLAMF7⁻ DNT in the presence or absence of AOA treatment (100 µM) for 24 h *in vitro*. (**K-L**) Glutamine uptake **K** and ATP levels **L** in SLAMF7^-^ and SLAMF7^+^ DNT after addition of V9302, an inhibitor of SLC1A5. (**M-N**) Flow cytometric analysis of the expression of Ki67 **M** an GZMB **N** in SLAMF7^-^ and SLAMF7^+^ DNT after addition of V9302

The genes critical for glutamine metabolism and transportation were analyzed using RNA-seq results, of which four genes had marked changes between SLAMF7^−^ and SLAMF7^+^ DNT, including aminotransferase *Gpt2* and *Got2*, transporters *Slc1a5* and *Slc38a1* (Fig. 6A). Then, we validated the expression of these four genes by qRT-PCR, and *Gpt2* and *Slc1a5* had the most pronounced changes (Fig. 6B). We further detected the expressions of GPT2 and SLC1A5 at protein level in DNT, respectively. Both of them expressed at higher levels in SLAMF7^+^ DNT than SLAMF7^−^ DNT, and downregulation of SLAMF7 expression reduced the GPT2 and SLC1A5 expression (Fig. 6C-F). Therefore, SLAMF7 could upregulate GPT2 and SLC1A5 expression in DNT.

In order to determine the role of GPT2 and SLC1A5 in mediating SLAMF7-driven glutamine metabolism and antitumor function in DNT, the corresponding inhibitors AOA and V9302 were used. To validate inhibitor specificity and establish optimal dosing for subsequent experiments, we conducted dose-response assays in DNT (Figure S3A). The concentrations that yielded definitive inhibitory effects—100 µM for AOA and 10 µM for V9302—were consistent with their previously reported effective doses [26, 27], corroborating the functional relevance of these concentrations. Furthermore, as shown in Fig. 6G-I and Figure S3B, inhibiting GPT2 by AOA significantly reduced the expression of proliferative protein Ki67, anti-apoptotic protein BCL2 and cytotoxic protein Perforin in SLAMF7^+^ DNT, whereas had no effect on SLAMF7^−^ DNT, suggesting that GPT2 activity is functionally required for SLAMF7-dependent cytotoxicity in DNT. We next assessed whether GPT2-mediated glutamine metabolism is required for the superior antitumor activity of SLAMF7⁺ DNT. In a direct tumor-killing assay, SLAMF7⁺ DNT demonstrated significantly greater lysis of A20 cells than SLAMF7⁻ DNT (Fig. 6J). Strikingly, inhibiting GPT2 with AOA severely compromised the cytotoxicity of SLAMF7⁺ DNT while having a minimal effect on SLAMF7⁻ DNT. Corresponding analysis confirmed that the SLAMF7-driven antitumor capacity was significantly more sensitive to AOA treatment (Figure S3B), establishing glutamine metabolism as a key metabolic pathway underlying SLAMF7-enhanced DNT function.

Additionally, inhibition of SLC1A5 by V9302 markedly decreased the level of glutamine uptake and ATP production in SLAMF7^+^ DNT, whereas it had no significant effect on SLAMF7^−^ DNT (Fig. 6K-L, and Figure S3C). The expression of Ki67 and cytotoxicity-related granzyme B were reduced by V9302 in SLAMF7^+^ DNT, but had no changes in SLAMF7^−^ DNT (Fig. 6M and N). These results suggested that the enhanced functional state of SLAMF7⁺ DNT is dependent on GPT2/SLC1A5-mediated glutamine metabolism, identifying both GPT2 and SLC1A5 as key downstream effectors of SLAMF7-driven metabolic reprogramming in DNT-mediated antitumor immunity.

### SLAMF7 activates ERK signaling pathway to target SLC1A5 and GPT2

**Fig. 7:**
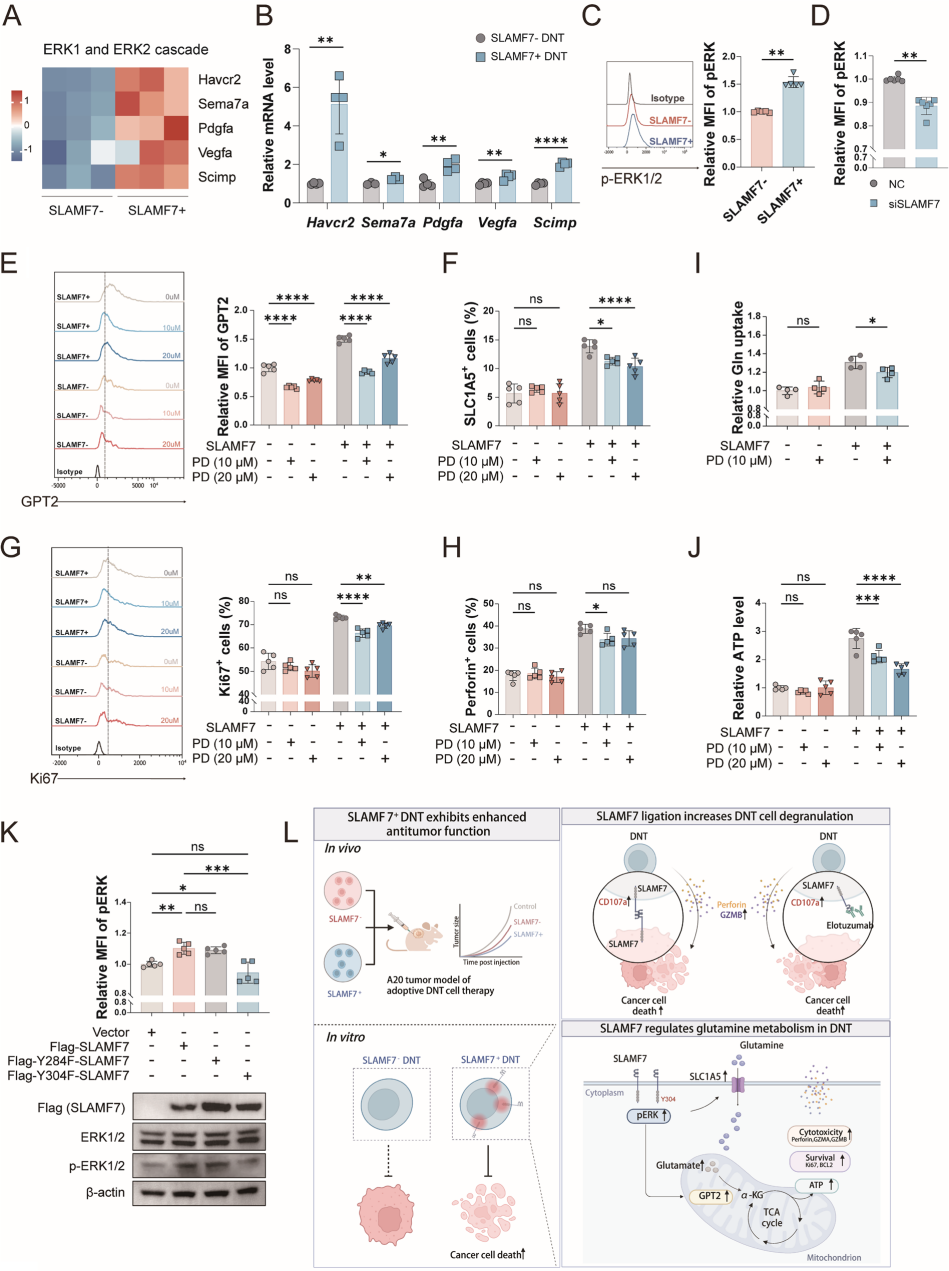
SLAMF7 regulates ERK signaling to target SLC1A5 and GPT2. (**A-B**) Heatmap of ERK1/2 cascade-related DEGs in SLAMF7^+^ DNT compared to SLAMF7^-^ DNT using RNA-seq data **A** and validated by qRT-PCR **B**. (**C-D**) Flow cytometric analysis of the expression of p-ERK1/2 in SLAMF7^-^ and SLAMF7^+^ DNT **C** or in siNC and siSLAMF7 DNT **D**. (**E-H**) Flow cytometric analysis of the expression of GPT2 **E**, SLC1A5 **F**, Ki67 **G** and Perforin **H** in SLAMF7^-^ and SLAMF7^+^ DNT after addition of PD98059, an inhibitor of p-ERK1/2. (**I-J**) Glutamine uptake **I** and ATP levels **J** in SLAMF7^-^ and SLAMF7^+^ DNT after addition of PD98059. **K** Flow cytometric analysis of p-ERK levels in GFP⁺ HEK293T cells, and Western blot analysis of total ERK1/2 and phosphorylated ERK1/2 in HEK293T cells transfected with vector control, Flag-tagged wild-type SLAMF7, Y284F mutant, or Y304F mutant. **L** A schematic illustration of SLAMF7 enhancing the efficacy of DNT-based adoptive cell therapy in cancer treatment

Previous study has shown that glutamine uptake and metabolism in T cells are regulated by extracellular signal regulated kinase (ERK) activity [28]. The ERK pathway is considered the key signaling pathway downstream of SLAMF7 [29]. In line with this, GO analysis revealed that ERK1/2 cascade and MAP kinase activity were significantly enriched in SLAMF7^+^ DNT (Figs. 5A and 7A). We therefore investigated whether ERK signaling might regulate glutamine metabolism in DNT by targeting the aminotransferase GPT2 and the glutamine transporter SLC1A5.

We first validated ERK signaling-related DEGs identified by GO enrichment analysis using qRT-PCR (Fig. 7A and B). Then we assessed the level of ERK phosphorylation (pERK), and found that it was significantly higher in SLAMF7^+^ DNT than SLAMF7^−^ DNT (Fig. 7C). Besides, downregulation of SLAMF7 expression reduced the level of pERK protein in DNT (Fig. 7D), revealing that SLAMF7 regulated ERK signaling in DNT. Furthermore, we blocked ERK activation by PD98059 in DNT with or without SLAMF7 expression to detected the expression of GPT2 and SLC1A5, the expression of functional molecules, and the level of glutamine metabolism. The results showed that blocking ERK activation had a downregulatory effect on the expression of GPT2 (Fig. 7E) and SLC1A5 (Fig. 7F), the expression of Ki67 and perforin (Fig. 7G and H), the level of glutamine uptake (Fig. 7I) and ATP production (Fig. 7J), which were more pronounced when SLAMF7 expression was present in DNT. Corresponding inhibition rate analysis were shown in Figure S3D. These findings indicated that SLAMF7 activates the ERK signaling pathway to upregulate the expression of SLC1A5 and GPT2, thereby promoting glutamine uptake and metabolism to support enhanced survival and cytotoxicity in DNT.

We sought to determine if SLAMF7 directly activates ERK signaling through its intracellular domain. Previous studies have indicated that Tyr304 is critical for its activating function and Tyr284 for its inhibitory role [30], Therefore, we created loss-of-function SLAMF7 mutants by substituting tyrosine with phenylalanine at these conserved intracellular residues (Y284F and Y304F). All constructs, including wild-type SLAMF7 and a vector control, were overexpressed in HEK293T cells (Fig. 7K and S3E). Analysis of GFP⁺ cells by flow cytometry demonstrated that wild-type SLAMF7 significantly boosted ERK phosphorylation. Importantly, the Y304F mutant lost this ability, while the Y284F mutant phenocopied the wild-type, still enhancing phosphorylation. These observations were validated at the protein level by western blot analysis (Fig. 7K). These data establish that SLAMF7 directly activates ERK by engaging the pathway through its intracellular Tyr304 residue.

## Discussion

Adoptive DNT cell therapy has emerged as a promising strategy in cancer immunotherapy due to its potent cytotoxic function and lack of GVHD [5, 7]. A thorough investigation of the underlying molecular mechanisms regulating DNT antitumor activity is crucial to identify novel therapeutic targets. In this study, we identify SLAMF7 as a critical regulator of DNT-mediated antitumor activity and a potential therapeutic target to enhance DNT-based immunotherapy.

Consistent with prior report [31], our study confirmed that SLAMF7 is highly expressed in CD8⁺ T cells and DNT in T cell subsets isolated from healthy controls’ peripheral blood. Our results further showed that SLAMF7 expression positively correlates cytotoxic and survival-related molecules in murine and human DNT, and it can enhance DNT antitumor activity both *in vitro a*nd *in vivo*. Based on these results, modulating their function via SLAMF7 may represent a promising strategy for clinical cancer immunotherapy.

In the tumor microenvironment (TME), the function of immune cells is not only determined by their intrinsic state but also regulated by ligand-receptor interactions. SLAM family receptors, including SLAMF7, are reported mostly self-ligands and engage in homotypic interactions [32]. SLAMF7-SLAMF7 interactions between macrophages and tumor cells are required for tumor cell phagocytosis [13, 17]. Additionally, SLAMF7 expression on tumor targets has been reported to enhance NK cell-mediated cytotoxicity via SLAMF7 ligation-induced degranulation [16, 33, 34]. In the current study, we demonstrated that SLAMF7 ligation with ligands of tumor cells or clinical-grade monoclonal antibody elotuzumab could enhance antitumor cytotoxicity in DNT by promoting cell degranulation. Importantly, SLAMF7 expression varies across tumor types, which might impact the broader applicability of SLAMF7-targeted DNT therapy. High levels of SLAMF7 were reported in hematologic malignancies such as chronic lymphocytic leukemia, diffuse large B-cell lymphoma, multiple myeloma, and myelodysplastic syndromes [13, 35]. These tumor types may be particularly susceptible to SLAMF7-based immunotherapies. Furthermore, our analysis of paired tumor and adjacent normal samples from the TCGA dataset revealed significantly elevated SLAMF7 expression in tumor tissues of head and neck squamous cell carcinoma (HNSC), kidney renal clear cell carcinoma (KIRC), kidney renal papillary cell carcinoma (KIRP), and lung adenocarcinoma (LUAD) compared to their adjacent normal tissues (Figure S2D). Although differential expression was observed in a limited number of tumor types, these findings supported the potential of SLAMF7 expression as a predictive biomarker to identify patients most likely to benefit from SLAMF7⁺ DNT therapy. These observations warrant further clinical validation.

Furthermore, we found that SLAMF7 also promotes DNT function in a ligand-independent manner, and this effect is closely associated with glutamine metabolism. Glutamine metabolism represents a major pathway regulating energy metabolism in T cells, supporting both their survival and effector functions [36, 37]. Effector T cells could increase the intake of glutamine to proliferate rapidly and promote the secretion of cytokines [38, 39]. Consistent with these findings, we found that SLAMF7⁺ DNT exhibit enhanced glutamine uptake and increased ATP production, accompanied by upregulation of glutamine transporter SLC1A5 and aminotransferase GPT2, two essential regulators of glutamine uptake and α-KG production [25, 40]. Conversely, knockdown of SLAMF7 significantly reduced cytotoxicity or ATP production in DNT. These findings preliminarily implied that SLAMF7 enhances the metabolic fitness of DNT by upregulating glutamine uptake and metabolism.

Previous studies have shown that ERK signaling regulates glutamine uptake and metabolism in T cells [28, 36]. Moreover, SLAMF7 has been previously implicated in ERK pathway activation in NK cells [29]. In line with this, our results demonstrated that SLAMF7 promotes the expression of SLC1A5 and GPT2 in an ERK-dependent manner, thereby supporting DNT survival and cytotoxic function. In the TME, competition for glutamine between tumor and immune cells leads to glutamine deficiency, which affects the function of immune cells [27, 36]. In addition, the activation of ERK signaling pathway was reported to upregulate glutamine uptake not only in T cells, but also in tumor cells [28, 41]. Therefore, regulation of ERK and glutamine metabolism-related genes by SLAMF7 may provide SLAMF7^+^ DNT with a metabolic advantage under glutamine-limited conditions, allowing sustained energy generation and effector molecule production. This mechanism implied that modulating the SLAMF7-ERK-glutamine metabolism axis could be a promising therapeutic strategy to boost adoptive DNT-based immunotherapy.

All human DNT experiments in this study were conducted using PBMC from healthy donors, which represents a limitation. However, healthy allogeneic DNT, including genetically modified DNT, have shown clinical safety and efficacy in early-phase trials for relapsed leukemia and B-cell lymphoma [7, 42, 43], supporting the feasibility of using ex vivo-expanded healthy DNT as a clinically applicable “off-the-shelf” cell therapy. These findings highlight the therapeutic relevance of our *in vitro* results. In parallel, emerging evidence has also reported that DNT from cancer patients exhibit reduced frequency and cytotoxicity in immunosuppressive tumor microenvironment [44]. Although this may limit the utility of autologous DNT in cancer patients, it raises the potential for DNT as biomarker for tumor progression or host immune status. Future studies will be necessary to profile SLAMF7 expression and metabolic programs in patient-derived DNT, and to evaluate whether the therapeutic mechanisms identified here are preserved or altered in cancer settings.

In conclusion, our study reveals a novel mechanism by which SLAMF7 not only intrinsically enhances DNT antitumor function by regulating ERK-GPT2/SCL1A5-mediated glutamine metabolic pathway, but also engages in homotypic ligand-receptor interactions with SLAMF7-expressing tumor cells to promote DNT cell degranulation, thereby exerting a dual role in antitumor immunity. Our findings present a new insight into the underlying antitumor mechanism of DNT and establish SLAMF7 as a new target for improving adoptive DNT-based cancer immunotherapy.

## Supplementary Information

Supplementary Material 1.

## Data Availability

The high-throughput sequencing data reported in this work were uploaded to the NCBI Sequence Read Archive (SRA) database under accession number PRJNA1276496.
