# Supplementary material for: SLAMF7 promotes TCRαβ+ double negative T cell antitumor activity through enhancing glutamine metabolism
Source: J Exp Clin Cancer Res. 2025 Oct 30;44:297. doi: 10.1186/s13046-025-03570-w (PMC12574265; doi:10.1186/s13046-025-03570-w)
Supplement: Supplementary file 1 — Supplementary Material 1. [file 13046_2025_3570_MOESM1_ESM.pdf]

Supplementary Figures and Tables

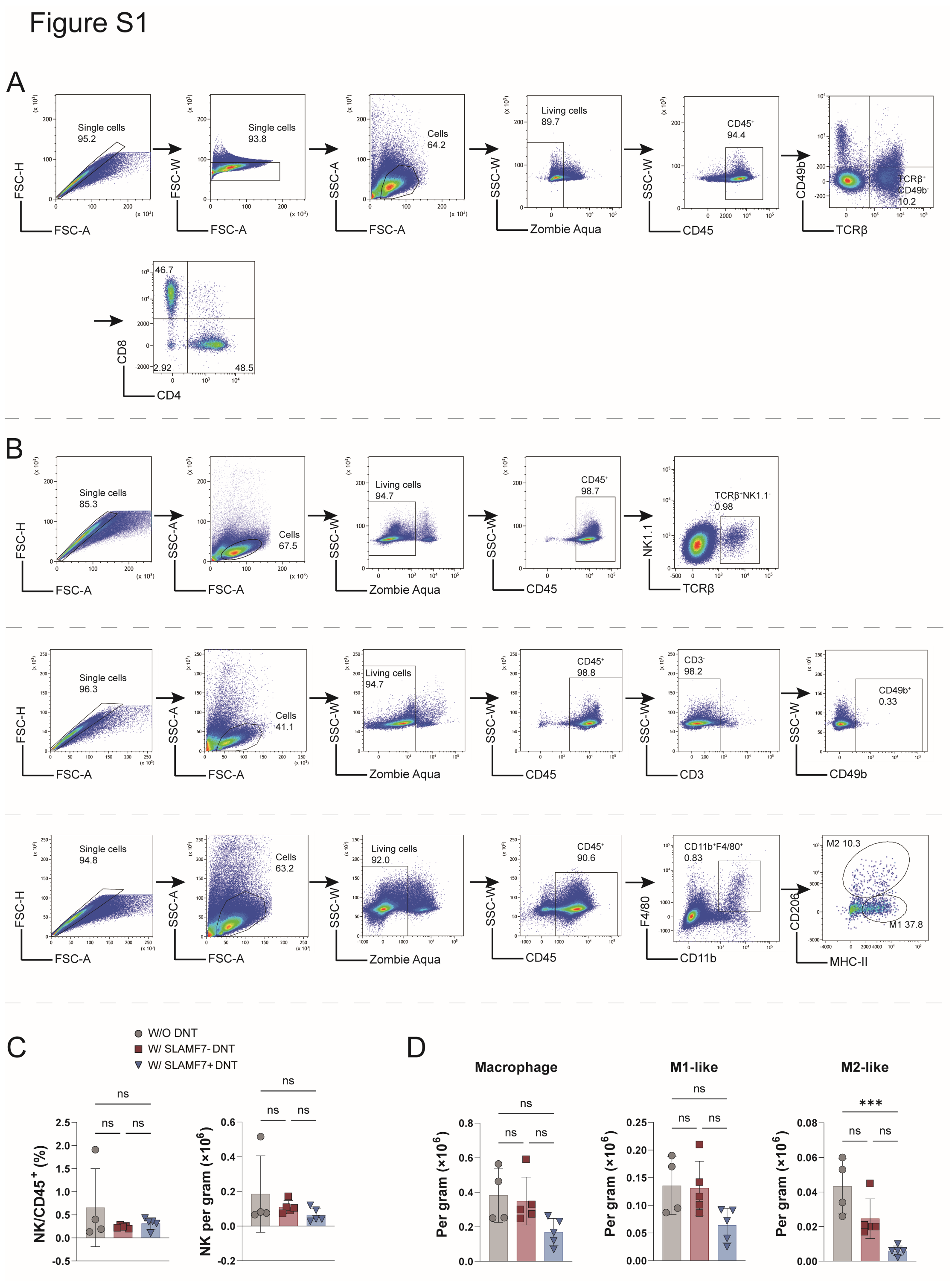

Figure S1. Flow cytometry gating strategy and profiling of tumor-infiltrating innate immune cells following DNT therapy. (A) Gating strategy for DNT (TCRβ^{+}CD49b^{-}CD4^{-}CD8^{-}) from tumor in the A20-bearing BALB/c mouse model as

shown. (B) Gating strategy for transferred-DNT (TCRβ^{+}NK1.1^{-}, from C57BL/6), tumor-infiltrating NK (CD3^{-}CD49b^{+}) and macrophages (CD11b^{+}F4/80^{+}; M1-like: CD11b^{+}F4/80^{+}MHC-II^{+}CD206^{-}; M2-like: CD11b^{+}F4/80^{+}MHC-II^{-}CD206^{+}) in the immunodeficient BALB/c nude mice bearing A20 tumor as shown. (C) Flow cytometric analysis of NK cell frequency and absolute number in tumors from BALB/c nude mice treated with SLAMF7^{+} or SLAMF7^{-} DNT, or without DNT. (D) Quantification of tumor-associated macrophages, including total, M1-like, and M2-like subsets per gram of tumor tissue.

Figure S2

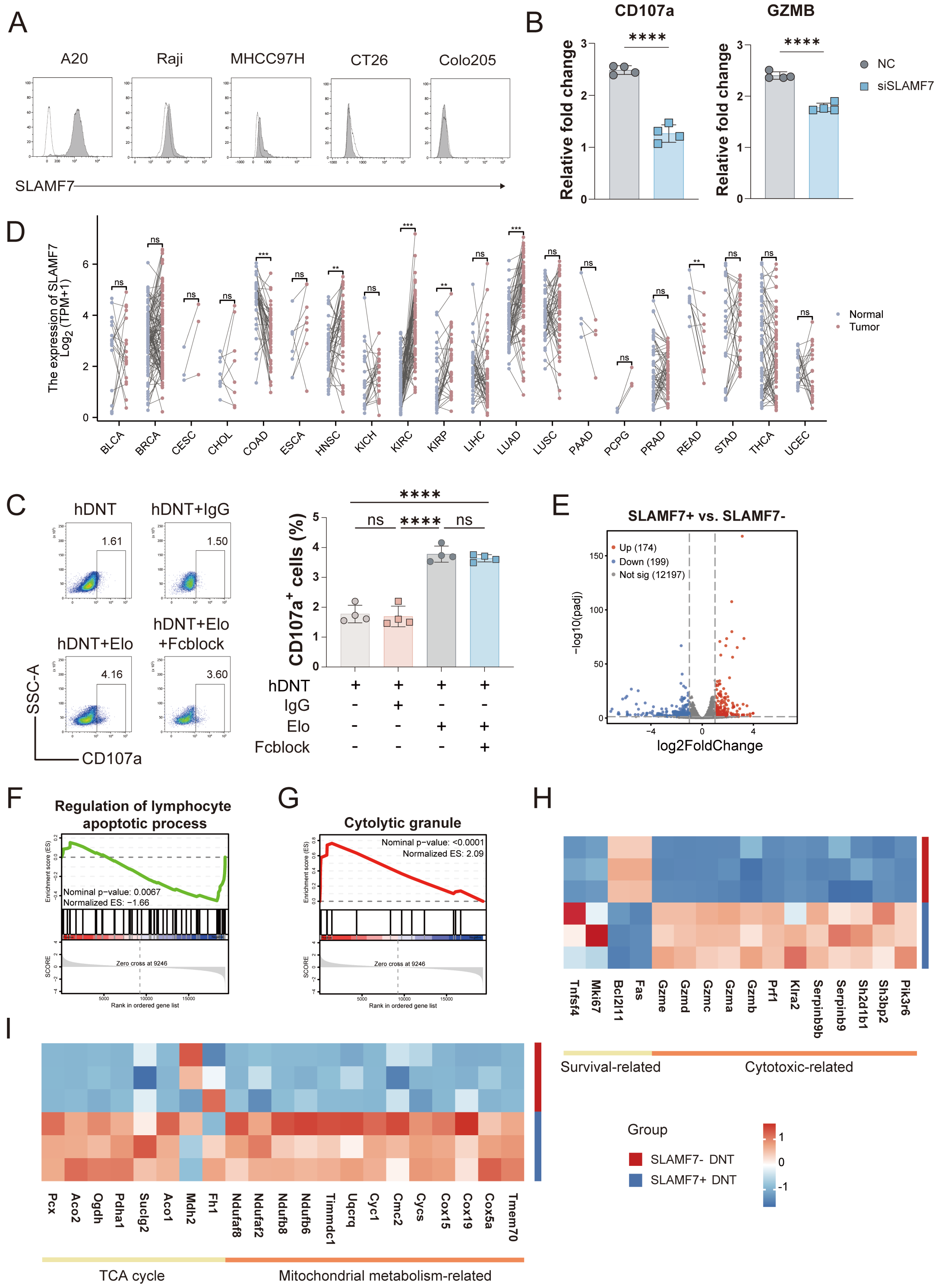

Figure S2. SLAMF7 ligation and transcriptome sequencing results. (A) The expression of SLAMF7 in each cell lines. (B) Relative fold change (calculated as: Value $_{\text{with anti-SLAMF7}}$ / Value $_{\text{without anti-SLAMF7}}$) of CD107a at cell surface and GZMB in control

and SLAMF7-knockdown DNT. (C) Representative flow cytometry plots and statistical analysis of percentage of CD107a$^+$ DNT cells following treatment with PBS, isotype IgG1, elotuzumab, or elotuzumab plus Fc receptor blocking reagent. (D) SLAMF7 expression across paired tumor and adjacent normal tissues from the TCGA dataset. (E) Distributions of DEGs in SLAMF7$^+$ and SLAMF7$^-$ DNT are demonstrated in a volcano plot. (F-G) GSEA enrichment analysis of lymphocyte-apoptotic process-related genes (F) and cytolytic granule-related genes (G) between SLAMF7$^+$ and SLAMF7$^-$ DNT. (H-I) The expression patterns of survival/cytotoxic-related genes (H) and TCA cycle/Mitochondrial metabolism-related genes (I) were confirmed by qRT-PCR.

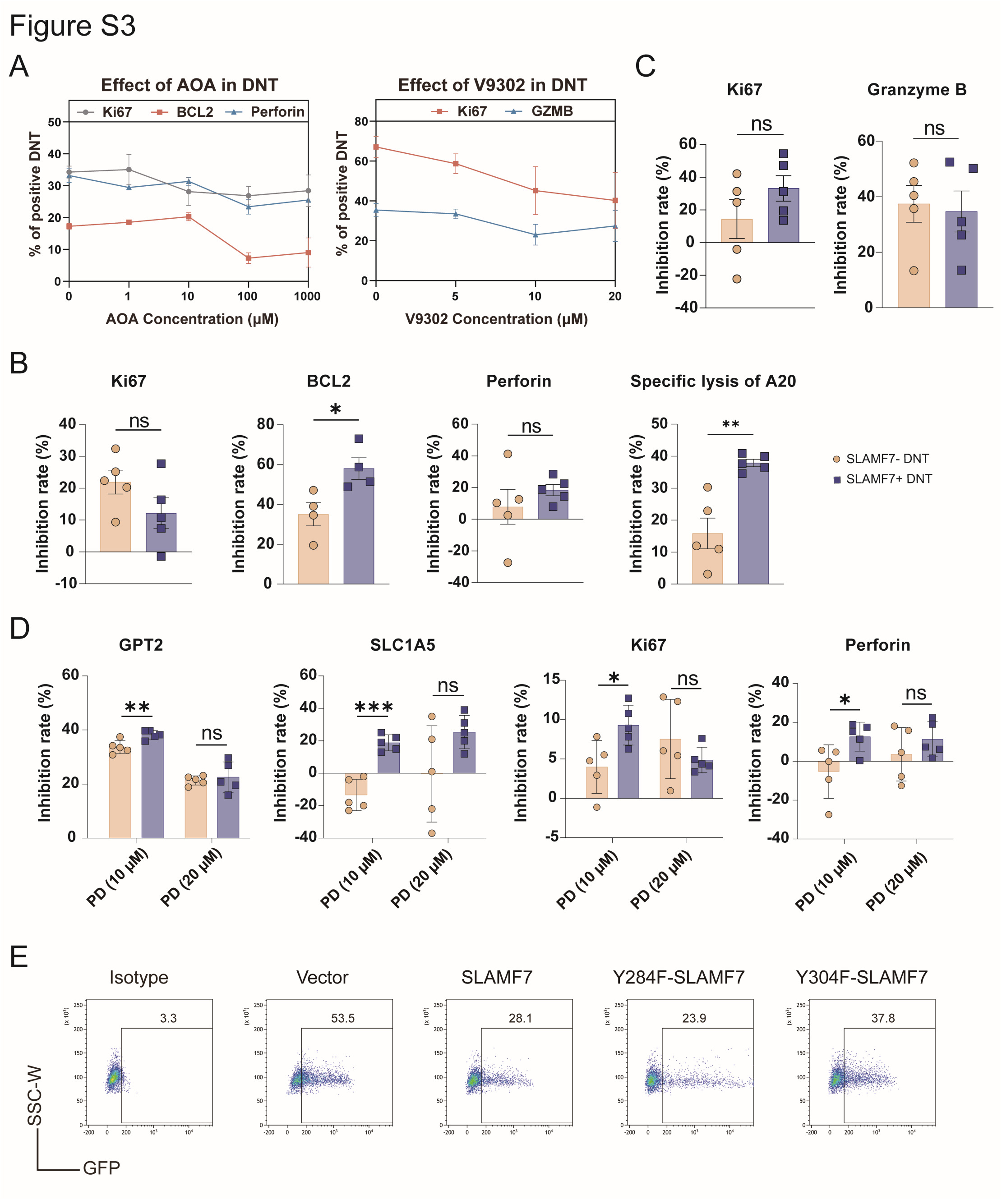

Figure S3. Dose-response analysis of inhibitors in DNT, and functional consequence of inhibitors' treatment. (A) DNT were treated with increasing concentrations of AOA (0-1000 μM) or V9302 (0-20 μM), and the expression levels of survival and cytotoxic moleculars were analyzed by flow cytometry. (B) Inhibition rate (calculated as: 100 × (value$_{\text{without inhibitor}}$ - value$_{\text{with inhibitor}}$) / value$_{\text{without inhibitor}}$) of Ki67, BCL2, Perforin and specific lysis of A20 in SLAMF7$^-$ and SLAMF7$^+$ DNT with addition of AOA. (C) Inhibition rate of Ki67 and GZMB in SLAMF7$^-$ and SLAMF7$^+$

DNT with addition of V9302. (D) Inhibition rate of GPT2, SLC1A5, Ki67 and Perforin in $SLAMF7^-$ and $SLAMF7^+$ DNT with addition of PD98059. (E) Representative flow cytometry plots of GFP^+ cells in HEK293T cells transfected with vector control, Flag-tagged wild-type SLAMF7, Y284F mutant, or Y304F mutant.

Supplementary Table 1. List of sequences used in the study.

Primer sequences used for real-time PCR

Species	Gene	Strand	Primer sequence (5'-3')
mouse	*Slamf7*	Sense	AAAAAGACGGCGTTACATCACA
		Antisense	GGCTGAGCTTCATGGAGTAGA
mouse	*Lamp1*	Sense	CCAGGCTTTCAAGGTGGACAGT
		Antisense	GGTAGGCAATGAGGACGATGAG
mouse	*Cd69*	Sense	GGGCTGTGTTAATAGTGGTCCTC
		Antisense	CTTGCAGGTAGCAACATGGTGG
mouse	*Gzmb*	Sense	CAGGAGAAGACCCAGCAAGTCA
		Antisense	CTCACAGCTCTAGTCCTCTTGG
mouse	*Prf1*	Sense	ACACAGTAGAGTGTCGCATGTAC
		Antisense	GTGGAGCTGTTAAAGTTGCGGG
mouse	*Mki67*	Sense	GAGGAGAAACGCCAACCAAGAG
		Antisense	TTTGTCCTCGGTGGCGTTATCC
mouse	*Gzma*	Sense	GTGGTGGAAAGGACTCCTGCAA
		Antisense	GAGAGGAAAGTATAGACACCAGG
mouse	*Gpt2*	Sense	CATTGCGGCAAGCCAAAGACCA
		Antisense	GCTTCTCTTCCCAGGCAAAGTG
mouse	*Slc1a5*	Sense	CTGCCTGTGAAGGACATCTCCT
		Antisense	CTCGGCATCTTGGTTCGATCCA
mouse	*Got2*	Sense	GCGGTTTTGACTTCTCTGGAGC
		Antisense	ACGGACGCTATCTCCTTCCACT
mouse	*Slc38a1*	Sense	TACCAGAGCACAGGCGACATTC
		Antisense	ATGGCGGCACAGGTGGAACTTT
mouse	*Havcr2*	Sense	TCAGGTCTTACCCTCAACTGTG
		Antisense	GGCATTCTTACCAACCTCAAACA
mouse	*Sema7a*	Sense	CATCGCTCAAAGGCTACCACATG
		Antisense	GGTGACTATCAGCTACCTGGAAG
mouse	*Pdgfa*	Sense	CTGGCTCGAAGTCAGATCCACA
		Antisense	GACTTGTCTCCAAGGCATCCTC
mouse	*Vegfa*	Sense	CTGCTGTAACGATGAAGCCCTG
		Antisense	GCTGTAGGAAGCTCATCTCTCC
mouse	*Scimp*	Sense	GCCCTCAAAACAGGATGGAAGAG
		Antisense	GGTTGTCTTGGAGCTTCCTGTG
mouse	*Tnfsf4*	Sense	GGAAGAAGACGCTAAGGCTGGT
		Antisense	CTGGTAACTGCTCCTCTGAGTC
mouse	*Bcl2l11*	Sense	GGAGATACGGATTGCACAGGAG

		Antisense	CTCCATACCAGACGGAAGATAAAG
mouse	*Fas*	Sense	CTGCGATTCTCCTGGCTGTGAA
		Antisense	CAACAACCATAGGCGATTTCTGG
mouse	*Gzme*	Sense	GAGATACTGTGGAGGCTTCTTGG
		Antisense	GGATGATCTGCTGTGTCTCCTC
mouse	*Gzmd*	Sense	CTGTGGAGGCTTCCTGATTCAAG
		Antisense	GGATGATCTGCTGTGTCTCCTC
mouse	*Gzmc*	Sense	AGATGTTCTGCGGAGGCTTCCT
		Antisense	GGATGATCTGCTGTGTCTCCTC
mouse	*Klra2*	Sense	CTGGATTGCACTGACACATCACG
		Antisense	GCACGGTCATCCTCTTCTGTAG
mouse	*Serpinb9b*	Sense	AAGGGATGTGGGCTTGCCAGTT
		Antisense	CTGGCAACTCATCCACAAAGGC
mouse	*Serpinb9*	Sense	AGAAGCAGAGGTGTCCAGGCAA
		Antisense	GGCATTGATGAGAACCAGCCTG
mouse	*Sh2d1b1*	Sense	AAGGGAGGTGTGGATGGCAACT
		Antisense	TCGTTCTTGGAGTATGAGCATTAG
mouse	*Sh3bp2*	Sense	GCTGCCTAACTCGGTGTTTGTC
		Antisense	CCTGACTTGGTAGAGGAGTTCC
mouse	*Pik3r6*	Sense	CCTACATCACCTTCCACCTCTG
		Antisense	CAAGGCATCCAGATCAGCACTG
mouse	*Tmem70*	Sense	AGTTGATAAGCCAGAAAATGGCAG
		Antisense	GAGAGAAGGTACGGCAAGAATGC
mouse	*Cox5a*	Sense	GTCACACGAGACAGATGAGGAG
		Antisense	CCGTCTACATGCTCGCAATGCA
mouse	*Cox19*	Sense	GACCGCAATGAACTTCGGGA
		Antisense	TCCATTAGGTCTCTAAAGCCGAG
mouse	*Cox15*	Sense	TCACGTCTGTCACTGCCATTA
		Antisense	GGGACTCTTCGGAGTTCATTCA
mouse	*Cycs*	Sense	GAGGCAAGCATAAGACTGGACC
		Antisense	ACTCCATCAGGGTATCCTCTCC
mouse	*Cmc2*	Sense	GTTTTTCGGCCATTGCAACGACC
		Antisense	GTCAGAAAGCCTCCGTCGCATG
mouse	*Cyc1*	Sense	CCATCTACACAGAAGTCTTGGAG
		Antisense	GCGTTTTCGATGGTCATGCTCTG
mouse	*Uqcrq*	Sense	CCTACAGCTTGTCGCCCTTT
		Antisense	GATCAGGTAGACCACTACAAACG

mouse	*Timmdc1*	Sense	ACGAGCAGAGGCTGGAAGAGTG
		Antisense	AGGCTTAGCAGCTCCTCAATCC
mouse	*Ndufb6*	Sense	CTCCAGTCTCTTCGCTGTTTCTC
		Antisense	CTGGGCTTCGAGCTAACAATGG
mouse	*Ndufb8*	Sense	CGCCAAGAAGTATAACATGCGAG
		Antisense	CCTCTCATGCTGTGATCGGTTG
mouse	*Ndufaf2*	Sense	GGATTGTAGAAGCAGCGAACAGA
		Antisense	TCCATAGTGGGTGGAGTCTTCC
mouse	*Ndufaf8*	Sense	CTCGGCTTATGGCAAGTGCGTG
		Antisense	CCTCCCATCATGGTCTTCTTGG
mouse	*Fh1*	Sense	GAATGGCAAGCCAAAATTCCTT
		Antisense	CGTTCTGTAGCACCTCCAATCTT
mouse	*Mdh2*	Sense	TCACTCCTGCTGAAGAACAGCC
		Antisense	CCTTTGAGGCAATCTGGCAACTG
mouse	*Aco1*	Sense	AGAACCCATTTGCACACCTTG
		Antisense	AGCGTCCGTATCTTGAGTCCT
mouse	*Suclg2*	Sense	AGCTCAAGGTGCCACTGGTAGT
		Antisense	GCTTTCTTGGCTGCATCCTCCA
mouse	*Pdha1*	Sense	GAAATGTGACCTTCATCGGCT
		Antisense	TGATCCGCCTTTAGCTCCATC
mouse	*Ogdh*	Sense	GGTGTCGTCAATCAGCCTGAGT
		Antisense	ATCCAGCCAGTGCTTGATGTGC
mouse	*Aco2*	Sense	ATCGAGCGGGGAAAGACATAC
		Antisense	TGATGGTACAGCCACCTTAGG
mouse	*Pcx*	Sense	GGATGACCTCACAGCCAAGCAT
		Antisense	GCAATCGAAGGCTGCGTACAGT
human	*Lamp1*	Sense	CGTGTCACGAAGGCGTTTTCAG
		Antisense	CTGTTCTCGTCCAGCAGACACT
human	*Cd69*	Sense	GCTGGACTTCAGCCCAAAATGC
		Antisense	AGTCCAACCCAGTGTTCCTCTC
human	*Gzmb*	Sense	CGACAGTACCATTGAGTTGTGCG
		Antisense	TTCGTCCATAGGAGACAATGCCC
human	*Prf1*	Sense	ACTCACAGGCAGCCAACTTTGC
		Antisense	CTCTTGAAGTCAGGGTGCAGCG
human	*Mki67*	Sense	GAAAGAGTGGCAACCTGCCTTC
		Antisense	GCACCAAGTTTTACTACATCTGCC

Sequences of the siRNA

Name	Forward (5'-3')	Reverse (5'-3')
siSLAMF7-1	CGGCGUUACAUCACAAAGUTT	ACUUUGUGAUGUAACGCCGTT
siSLAMF7-2	CUGCAUGUCUACAAGCAUUTT	AAUGCUUGUAGACAUGCAGTT
